# Around the Hospitals

**Published:** 1896-09-26

**Authors:** 


### AROUND THE HOSPITALS.

[Notice to the Managers of Institutions.—Special space is now reserved for the insertion of notices of hospital meetings and festivals. The Editor requests that all notices may reach The Hospital Office, 428, Strand, at least one week before the date of each meeting.]

**Prince Alfred Hospital, Sydney.**—The annual report of the Prince Alfred Hospital contains many interesting items. Although the hospital only contains 236 beds, and although one of the large wards, containing 30 beds, was closed for some weeks while being thoroughly renovated, the large number of 3,251 in-patients were treated during the year, against 2,927 for 1894, and the number of operations performed was 1,327, an increase of 64 for the year. This is the largest number of operations performed at any similar Australasian hospital, showing that the Prince Alfred is keeping up its reputation for surgical work. Typhoid fever has been rather more prevalent in the colony during the past year, and 170 cases were treated in the wards of the hospital. The total amount contributed by patients during the year under review was £2,278, about £214 less than during the previous year. Subscriptions and donations amounted to £2,279, against £1,531 in 1894, whilst during the year £660 was received from the Hospital Saturday Fund. The total income for the year was £13,783, towards which the Government contributed £7,635. The average cost per occupied bed was £67 9s. 5d. The nursing staff is still keeping up its high standard of efficiency, under the able management of Miss McGahey, the energetic and popular matron. Dr. Sankins is still the resident medical superintendent, and has carried out the instructions of the board with marked ability and zeal.

**Carrington Convalescent Hospital, Camden, N.S.W.**—The Carrington Hospital shows an increase of work for the past year, 1,094 patients having been treated. The number of beds in the hospital is 95, and the daily average number of patients was 76, as against 74 in 1894. The average annual cost per occupied bed was £34 3s. 11d. The number of contributing patients was less, being 109, and the amount contributed was £161 17s. 5d. During the year the hospital sustained the loss of its vice-president, Mr. W. H. Paling, who was the founder of the institution, he during the prime of an active life having presented to the colony on its centennial anniversary this magnificent gift for the benefit of the poor, the suffering, and the needy. The donation consisted of a beautifully situated estate of 450 acres with its improvements, together with a cheque for £10,000. It is situate in the Camden district, 42 miles from Sydney, and is approached by railway to within two miles of the hospital buildings. Sir Alfred Roberts, who has been the hon. secretary since the inception of the hospital, has found it necessary, owing to ill-health, to resign that office, and he has been unanimously elected to the position of vice-president. The office of hon. secretary has been filled by the appointment of two of the directors, Mr. E. R. Deas-Thomson and Dr. Cecil Purser. Miss Mona Ker still occupies the position of matron, which post she fills ably and well.

**University College Hospital.**—The plan on which the rebuilding of this hospital is to be carried out, was decided on during the year 1895 and will involve the gradual building of a new hospital with accommodation for an additional 90 beds, *i.e.*, 300 in all. The funds at present at the disposal of the committee for building will only, however, at first admit of the erection of one wing of the new hospital, and the committee appeal for contributions to the building fund, which may be either paid at once or spread over any number of years as the donors prefer. The committee did not hold a festival dinner in 1895, but as the result of a special appeal £89 was received in annual subscriptions and £1,567 in donations. Besides this £51 was received in annual subscriptions, and £1,299 in donations in consequence of the Christmas appeal made in 1894. The total cost of appeals in 1895 was £452. While on the subject of finances it would be well to point out that at the end of 1895 the debt to bankers and tradesmen had reached £14,765, an increase of nearly £3,000 on the previous year. Of this sum £8,000 was due to bankers. One excellent feature of this hospital is the fact that there are no less than seven Samaritan funds for the aid of the patients, the combined receipts of which in 1895 reached £1,664, and the expenditure during the same period £1,659. Through one of these funds—the Yates Fund—surgical and other appliances are supplied to poor patients, chiefly outdoor. These cost altogether £624, towards which patients contributed £119. The other funds are chiefly utilised in sending patients to convalescent homes. During 1895 the medicinal baths in connection with the hospital were used by 2,217 free and 212 paying patients. In order to assimilate the practice at University College as regards the board of the resident medical officers with that of other institutions, the charge of £1 1s. a week made to each resident officer has been abolished. This will mean an additional expenditure on the part of the hospital of rather over £400 a year.

